# Role of Technological Knowledge and Entrepreneurial Orientation on Entrepreneurial Success: A Mediating Role of Psychological Capital

**DOI:** 10.3389/fpsyg.2021.814733

**Published:** 2021-12-22

**Authors:** Ben-Oni Ardelean

**Affiliations:** ^1^Baptist Theological Institute, Bucharest, Romania; ^2^Faculty of Baptist Theology, University of Bucharest, Bucharest, Romania

**Keywords:** entrepreneurship, technological knowledge, entrepreneurial orientation, psychological capital, entrepreneurial success

## Abstract

This study pursues to build the conceptual model of entrepreneurial success (ES) that discusses the concept and phenomenon of ES and its perquisites and outcomes. This proposed mode anticipated that factors technological knowledge (TK), entrepreneurial orientation (EO), and psychological knowledge influence ES. This paper explains previous literature on perquisites, the phenomenon of TK, EO and psychological knowledge, and ES. This conceptual paper targets the scholarly works that provide support for the proposed model. A significant contribution of this paper is to propose an original relationship between prerequisites, phenomena, and consequences in ES. The proposed model shows a novel conceptualization of how these constructs may be connected to affect ES outcomes. This study enhances the literature by providing the theoretical literature of forerunners and outcomes for ES. In addition, this study has important implications for practitioners and entrepreneurs to generate success in entrepreneurial activities. Based on new insights, this study also developed and suggested new approaches and opportunities for future research.

## Introduction

Over the decades, the progressing digital trend involving the role of advanced innovations has coordinated the emerging demand for technological solutions. The rapid innovative advancement has profoundly changed the business dynamics, significantly shaping the business models through IT solutions. The developing technology paradigm has enhanced the potential of advanced technologies, thereby vigorously modifying entrepreneurial activities (Elia et al., 2020). The rigorous diffusion of the modernized invention (i.e., Information Communication Technology) has upheld the modern roads for incorporating novel innovations in entrepreneurial success (ES).

Indeed, this relentless convergence between entrepreneurship and digital technologies has advanced the new breed of entrepreneurs by successfully launching the new venture. Developing entrepreneurship has potentially been emerged as a prime feature increasing the firms’ growth rate. The field of entrepreneurship supporting the new venture’s success has become critical for all organizations and individuals. Undoubtedly, every individual wants to be a successful entrepreneur but what makes the organization successful is still a mystery to many minds. Passion, conviction, resourcefulness, and willingness to innovate are strong determinants of succeeding as an entrepreneur.

The entrepreneurs’ efforts play a strategic role in firms’ success. The developing significance of venture start-ups has enhanced the business environment by empowering the business visionaries to relish the ventures’ success. The study of the new venture’s success has gained the great interest of entrepreneurs, fundamentally featuring growth in firms’ performance, economic development, and value creation (Urbano et al., 2020). Entrepreneurs improve the firm’s profits by generating benefits and gaining business success. Given the statement, the research shows that ES exclusively depends on firms’ performance, financial satisfaction, community impact, employee fulfillment, and knowledge acquisition (Wach et al., 2020).

Over the years, business entrepreneurship has fundamentally evolved, bringing fruitful results for world economies. Perhaps, in recent years, the literature reveals that developing nations are focusing more on optimizing their entrepreneurial practices. The optimization structure fundamentally accelerates the nation’s economic growth. The study shows that nations like Dutch and Hungarian have revealed a healthy economic and social environment where entrepreneurial activities have flourished tremendously (Meyer and De Jongh, 2018). Regardless of whether it is Germany, China, or Russia, every country is relishing the advantage of entrepreneurship. The ES determinants [e.g., technological knowledge (TK), entrepreneurial orientation (EO), and psychological capital (PsyCap)] regulating the world’s economy make the firm grow through mergers and acquisitions, thus enabling the organizations to achieve long-term economic success (Sergi et al., 2019). Perhaps, understanding entrepreneurship, its processes, and perks compel the organizations in extending their entrepreneurial activities, ultimately gaining business success. Thus, it has become necessary for future researchers to inspect the critical success factors affecting the firms ’success (Gupta and Mirchandani, 2018).

Primarily, the technological developments modeled by strong learning capabilities enable the firms to achieve digital-centered ES. Technical knowledge refers to an individual’s knowledge regarding the use of technical advances (i.e., tools, devices, applications). It explains the entrepreneurs’ ability to raise innovative start-ups by commercializing the improvement of digital products (von Briel et al., 2018). Increasing TK guides the entrepreneurs to unleash the process of creative destruction, replacing the firms’ traditional products with innovative offerings. The progressing digital innovations produce knowledge that unfolds the firms to discover profitable alteration of the business resources. The emerging TK develops innovative products and services, positioning the firm to successfully compete with the competitors, thereby increasing the firm’s market share and overall success.

Entrepreneurship is the leading driver of business success, particularly integrating digital knowledge in firms’ business processes. The technical information potentially adds to the firm’s market value whereby establishing the modernized information network. In support, the study shows that the leading entrepreneurial ventures exploit the firms’ TK, thereby exploring the strategic opportunities in gaining business success (Lindholm-Dahlstrand et al., 2019).

The technical knowledge facilities the entrepreneurial start-ups. It improves business methods (Geissinger et al., 2019), potentially elucidating the firms’ innovations into business success (Tomy and Pardede, 2018). TK assists start-ups by incorporating digital information for achieving business success. The entrepreneurial venture having the highest knowledge proposition boosts the firms’ productivity. Given the statement, technical knowledge improves the efficiency of business processes by reengineering the conventional business models, thereby supporting successful business operations.

In addition, technological innovation underpins knowledge information by developing EO as a significant concept in entrepreneurship. EO refers to firms’ practices and activities driving the business innovation and market entry decisions (Covin and Wales, 2019). EO is a relevant phenomenon formulated around three entrepreneurial dimensions (i.e., technological innovations, pro-activeness, and risk-taking). EO innovation encourages the development of new products and services. At the same time, proactive behavior emphasizes seeking strategic opportunities with risk-taking behavior, demonstrating the individuals’ willingness to adapt to changing business environments. The entrepreneurial dimensions enhance the firms’ profitability and growth. These domains allow the firms to take advantage of strategic market opportunities, enabling the entrepreneurial venture to flourish for greatness. Perhaps, EO is a prime catalyst, recording the firms’ success. In recent times, research shows that EO has emerged as a fundamental concept in the management literature (Covin and Wales, 2019). Traditionally, entrepreneurship was considered the prime driver of business success, but now modern attention has been paid to the EO by various researchers.

The goal of the entrepreneurs is to drive the business operations, thus gaining ES. Nonetheless, to achieve the entrepreneur objective, EO plays a strategic role in improving the firms’ performance, thus gaining business success (Venkataraman, 2019). Consistently, the firms’ performance plays a crucial role in achieving business success. Literature shows that considerable attention had paid to the EO concerning business performance. In support, the result appears that EO improves business performance (Cho and Lee, 2018), subsequently accomplishing business objectives (i.e., ES).

Moreover, the psychological measure included within the EO enables the entrepreneurs to drive successful entrepreneurial activities. In entrepreneurship, the theoretical basis of psychological capital (PsyCap) refers to the individuals’ attitude toward entrepreneurial activities. PsyCap is a vital trait critically affecting the employees’ work behavior. PsyCap refers to an individual positive mental ability to perform the work task, ultimately gaining business success (Zhang et al., 2020). A healthy environment improves individuals’ capability to work and motivates them to achieve a higher degree of success. Given the statement, PsyCap positively influences individual well-being. In other words, PsyCap focuses on individual personal and professional development by improving their work performance (Tsai et al., 2020). The study illustrates that the PsyCap constructs (e.g., human, physical, tangible, and intangible resources) allow entrepreneurs to take advantage of the market opportunities (Ephrem et al., 2021), potentially making the firm stand out. Significantly, PsyCap drives the firm performance by building a critical relationship with ES (Paul and Tresita, 2018).

However, despite the increasing relevance of the research subject, little discussion has been recorded in the existing literature concerning the relationship of TK, EO, and PsyCap with ES. The study illustrates that entrepreneurial research influencing the knowledge environment has ignored the role of digital technology in ES (Elia et al., 2020). Similarly, the existing literature shows that little empirical research on EO (Rigtering et al., 2019) and physical capital had been dedicated to achieving business success (Paul and Tresita, 2018).

However, this study bridges the gap by identifying the number of the relevant determinants influencing the firms’ success. It potentially draws a simplified web of interrelated determinants affecting ES. The complexity explains that little research has been found on these variables, thus making the organization miss the strategic entrepreneurial opportunities.

Perhaps, this existing crevice has restrained the understanding of interdependencies between the components, driving the firm’s success. Hence, in entrepreneurship, there is a critical need for conceptualizing the following terms into entrepreneurial literature. In this regard, for getting considerable research understanding, TK, EO, and PsyCap need to be formally comprehended.

Altogether, this paper builds a theoretical foundation for understanding the role of TK in driving the firms’ success. From a theoretical perspective, the article determines the view of EO in the frame of ES. Furthermore, this research aims to examine the relationship of TK and EO with PsyCap. In this attempt to bridge the academic gap, the study questions the effect of firms’ digital innovation (i.e., TK) and EO under the mediating role of PsyCap on a firm’s success.

However, this study is essentially needed as it provides detailed information to the entrepreneurial community, thus making them aware of the impact of the determinants on firms’ success. This dominant approach of integrating the magnitude number of factors affecting ES increases the novelty of the proposed model. The study incorporates a novel theoretical model by systematically presenting the latest knowledge on the determinants of business success. The proposed model for the first time illustrates the mediating role of PsyCap under this context. Fundamentally, these changes make the suggested model to be different from the previous approaches.

In recent years, the COVID-19 pandemic has appeared like a disaster impacting the world economies (Parnell et al., 2020). The COVID-19 has devastatingly affected entrepreneurial operations, thus decreasing the overall firms’ success rate. Accordingly, literature shows that the pandemic has made entrepreneurs suffer badly. Entrepreneurs strategically benefit from market opportunities by working for society’s welfare. Indeed, this pandemic has made entrepreneurs deal with ongoing critical situations (Nasar et al., 2021), thus limiting business progress. Significantly, this paper provides the academic scholars with a clear understanding of how to contribute to the firm’s success, thereby overcoming the border of limitations. The technological aspect discussed in the study encourages the participating actors (i.e., stakeholder, management) to understand the role of technical knowledge.

Entrepreneurship is a phenomenon that drives economic growth while contributing to the firms’ success. It accelerates employment opportunities, innovation, thus stimulating the firms’ competitiveness (Crudu, 2019). Entrepreneurship reduces the risk and uncertainty, thereby enhancing the organizations’ productivity level. On the other hand, the disadvantaged entrepreneurs appear to harm society, thus decreasing the firms’ success rate.

## Literature Review

The increasing knowledge potential has received fundamental consideration in entrepreneurial research where its extended application had crossed the boundaries, driving technological advancement in entrepreneurship. Primarily, Section “Literature Review” presents significant reflections of various papers while proposing a modernized business model. The following section sheds light on the fundamental concepts, thus explaining some leading definitions in the light of the latest academic studies. The literature review aims to develop connections between the variables. Subsequently, the inherit section provides a comprehensive insight into the following terms; TK, ES, EO, and PsyCap. Indeed, all these variables are presented in the same sequence as illustrated above.

### Technological Knowledge and Entrepreneurial Success

Knowledge frontiers play an integral role in entrepreneurs’ life, centrally driving entrepreneurial activities. The knowledge boundary offers the venue to the venture capitalist, translating the firms’ strategic activities into ES. In today’s world of increasing competition, the organization aims to conquer the competition, thereby winning an advantage. Presently, the competitive environment has compelled businesses to innovate for generating novel digital competencies. In line with the statement, the study explains that TK has potentially collaborated with the market dynamic, thus translating entrepreneurial efforts into firms’ success (Dong, 2019).

Undoubtedly, the 21st-century advanced insurgencies have transformed the conventional business models, advancing the firms’ processes to incorporating modernized technological developments (for example, internet web, artificial intelligence, cloud computing). The advanced infrastructure develops superior value propositions by generating a high level of knowledge, technologically benefiting the firm (Camisón-Haba et al., 2019). Business success largely depends on the technological progression enabling the enterprise to seek sustainable growth (Sivam et al., 2019). In particular, the adaption of state-of-the-art- technologies (i.e., internet). (Omotosho, 2020) has suggested new ways of advancing business activities by recording an increase in firms’ success (Makiwa and Steyn, 2018).

Technological knowledge is a significant player in stimulating entrepreneurial progress. In recent years, entrepreneurs have valued the role of technical knowledge in accelerating business operations by winning strategic business opportunities. The recognition of entrepreneurial opportunities is an essential step in integrating technological computations in firms’ business operations. The extended range of entrepreneurial opportunities encourages entrepreneurs to produce and share technical information in this digital age. The successful applications of technologies enhance the organization’s resources, fundamentally improving the firms’ capabilities in gaining business success (Colomo-Palacios et al., 2018). The successful application of digital technologies allows entrepreneurs to leverage new market opportunities, allowing the firm to translate innovation into enhanced business knowledge (Rippa and Secundo, 2019). Given the articulation, the study states that due to the growing recognition of digitization, the organizations (i.e., entrepreneurial ventures) are rapidly transforming their practices, thereby gaining business success (Kuratko and Morris, 2018).

In particular, TK facilitates the firms’ performance, attaining organizational success in the shape of potential developments and business expansions (Bai et al., 2018). Technical knowledge constitutes the central requirement of the firm to innovate, encouraging the development of digital products. The digitalization proposed by the businesses forms a vital component in enhancing business knowledge, boosting entrepreneurial performance. Similarly, the research shows that TK enhances the business process, thus enhancing performance effectiveness (Akpan et al., 2020). Performance is a strong determinant of firm success. Given the statement, TK helps the firm in improving business performance, subsequently ensuring ES.

Indeed, the recent study states that the power of TK forms the basis of organizational activities, eventually manifesting the firms’ market position (Jakobsen et al., 2019). Entrepreneurs are increasingly adapting specialized methods for increasing the firms’ productivity. The generation of firms’ TK assists the entrepreneurs in enhancing the firms’ operations, thus increasing the firm’s productivity. Fundamentally, the inherited digital revolution has established a strong knowledge foundation, empowering entrepreneurs to expand their technical competencies, thus gaining business success. Hence, the literature found a positive relationship between TK and ES. Based on the theoretical support, this study proposed:

P1: Technological Knowledge is more positively related to ES.

### Entrepreneurial Orientation and Entrepreneurial Success

Today’s competitive environment has led businesses to face market uncertainties resulting in entrepreneurial failures. However, due to the increasing market vulnerability, it has become vital for emerging entrepreneurial organizations to sustain in this competitive environment of volatility. In such circumstances, EO has received substantial consideration while developing a cumulative relationship with business success.

The changing business environment has enforced EO to empower the firms to rapidly evolve business processes for succeeding in the world of fierce competition. The constructive effect of EO allows the entrepreneurs to embrace strategies in adjusting to the changing business environment. Business success in a highly violate environment views the EO as an integral factor driving the firms’ performance.

Entrepreneurial orientation is a prime concept encouraging firms to exhibit effective business performance under profoundly uncertain environments. EO is a strategic process that ensures the implementation of new business procedures. Indeed, the EO phenomenon makes firms meet the demands of the turbulent business environment. The EO leads the firms to adjust to changing business environments while rebuilding the conventional business processes through devising novel openings to maintain benefit. Given the articulation, the study states that EO enhances business performance (Covin and Wales, 2019), thereby gaining superior economic development.

Firms’ performance is a critical factor in driving business success. Literature suggests that the importance of business success not only depends on the firms’ tangible resources instead focuses on the development of human and technological skills. Hence, to fulfill the purpose, firms are rigorously adapting EO tendencies for achieving firms’ sustainability and growth (Erken et al., 2018). The findings suggest that entrepreneurs and managers should improve EO tendencies (i.e., innovativeness, pro-activeness, and risk-taking), thus gaining business success (Al-Samarraie et al., 2019).

Entrepreneurial orientation involves positive psychological traits (e.g., courage, risk-taking, pro-activeness), alleviating entrepreneurial performance. Firms’ performance is an essential predictor of ES. Following the statement, the study shows that EO positively influences the firm’s financial performance (Cho and Lee, 2018). The study illustrates that EO positively affects a firm’s innovation performance (Shaher and Ali, 2020). Further, the empirical evidence depicting the firm’s success found a significant relationship between EO and business performance (Cuevas-Vargas et al., 2019).

Indeed, EO alleviates the firms’ performance (Basco et al., 2019). The study shows that EO persuasively understands the market conditions, thus enhancing the organization’s performance (Khan et al., 2020). Significantly, the research shows that EO is a prime determinant of firms’ performance (Kittikunchotiwut, 2020). Hence, the findings have confirmed that EO owns the capability of enhancing the firms’ performance, thereby making the business ventures successful (Zaman et al., 2019).

In entrepreneurship, the concept of EO is at its boom, significantly advancing the business expansion (i.e., social and economic). In particular, research shows EO encourages the firms to acknowledge new technologies, innovation, and opportunities, thereby leading the organization to experience a successful entrepreneurial venture (Zaremohzzabieh et al., 2019). EO makes the organization act entrepreneurially, making the firms pursue new opportunities, significantly contributing toward business success.

Entrepreneurial orientation is a significant precedent in achieving business growth. As a result, firms are potentially adaptation the EO approach for promoting business success. Consistency, the literature suggests that firms’ practicing the entrepreneurial orientation experience an increase in business growth. Indeed, the literature proposes that EO is a strategic tool determining ES (Bernoster et al., 2020). Based on the theoretical support, this study proposed:

P2: Entrepreneurial orientation is more positively related to ES.

### Technological Knowledge and Psychological Capital

The expanding market competition and the emerging globalization have developed information knowledge as a strategic source of obtaining a competitive advantage. The firm’s psychological resource largely depends upon the creation of TK. PsyCap is a valuable concept that uses knowledge for enhancing the firms’ operations. Technical knowledge develops a critical resource for boosting the firms’ productivity. PsyCap forms a significant root in achieving positive outcomes (e.g., stat- of- the art knowledge). The emerging professional knowledge creates value for the firms’ resources, making the businesses generate new products. PsyCap plays an instrumental role in enhancing the firms’ innovation process. TK encourages firms to implement and generate new ideas. PsyCap in entrepreneurial ventures enhanced the individual mental capabilities, thus facilitating the utilization of digital applications. From the psychological perspective, the PsyCap polishes the skill set of the employees, thereby promoting technological progression and firms’ innovation.

Moreover, PsyCap is a unique concept, extending the role of human resources. The core competitiveness of digital knowledge enables the employees to construct their competency around the dimension of PsyCap (i.e., creativity), thus learning new technologies (Arasli et al., 2020). Good knowledge management not only improves the employees’ self-consciousness but records a triple down effect on individuals’ mental state, enhancing the individual’s PsyCap (Shen et al., 2019).

Perhaps, in this era of digitization, technological innovation is a prime factor contributing to firms’ long-term development. Digital knowledge enables individuals to alter their work behavior while optimally utilization the firms’ technologies. The advancement in digital technologies influences employees’ innovative behavior while promoting the creation of modern ideas. In conclusion, the research states that individuals’ behavior is positively associated with PsyCap (e.g., self-efficacy, risk-taking, resilience) (Sameer, 2018).

P3: Technological Knowledge is more positively related to PsyCap.

### Entrepreneurial Orientation and Psychological Capital

In entrepreneurship, understanding the role of human capital has become significantly essential in explaining the fundamental relationship of EO with PsyCap. This inherent relationship between the intellectual capital and entrepreneurs’ psychological wellness compels the businesses to enhance business performance. Entrepreneurial ventures do not operate on entrepreneurs’ wisdom only, but it also needs an individual’s psychological strength to overcome business uncertainties.

In recent years, the rapid development of innovation has enhanced the firms’ entrepreneurial activities while making the PsyCap play a significant role in economic development (Jiang et al., 2019). The study shows that venture capitalist plays an integral role in improving the firms’ innovative performance (Gu et al., 2018). As a result, entrepreneurs’ psychological state is a crucial component determining their performance. The characteristics of the entrepreneur (e.g., self-efficacy, pro-activeness, innovativeness, creativity) affect the firms’ performance. In support, the study shows that entrepreneurial competencies enhance the effect of personality traits (i.e., innovation, affection) on firms’ PsyCap (Hasan et al., 2019).

Indeed, this research topic is of great significance that suggests the need to realize the influence of EO on personality traits (i.e., PsyCap). Individuals having good psychological well-being are most likely to become successful entrepreneurs (Bailey et al., 2018). Perhaps, the dominant traits (e.g., self-confidence, optimum, and innovations) maximize the entrepreneurs’ knowledge while making the firm continually innovate, thus relishing the business earnings.

P4: Entrepreneurial Orientation is more positively related to the PsyCap.

### Psychological Capital and Entrepreneurial Success

The research argues that emerging psychological driver among entrepreneurs has compounding effects on ES (Bignotti and Le Roux, 2018). Successful entrepreneurial ventures provide a role model for future venture capitals to look up, get inspired, and initiate a new start-up journey in the violate business environment. Venture capitalists are the individuals that invest in the firms’ start-ups. Venture capitalists not only invest in the firm but also contributes additional resources for gaining superior performance. As a result, the study suggests that entrepreneurial enterprises need to innovate business activities to achieve higher business returns (Wang et al., 2019). In such circumstances, PsyCap provides valuable resources for entrepreneurs to succeed.

Psychological capital exhibiting positive organizational behavior refers to the individuals’ mental state, constructed across four attributes: hope, optimism, self-efficacy, and resilience. The promising result showed that the psychological element in entrepreneurial characteristics stimulates the firms’ performance (Gupta and Mirchandani, 2018), thereby increasing the entrepreneurs’ likelihood to succeed. In various literature, PsyCap is an essential determinant of the firm success. PsyCap enhances entrepreneurial competencies, thus boosting the firms’ productivity. In particular, examining the effect of PsyCap, the study reports that entrepreneurs high on resilience are most likely to succeed in their entrepreneurship (Baluku et al., 2018). Similarly, individuals high on PsyCap tend to exhibit positive behaviors (e.g., innovation, creativity), leading the entrepreneurs to experience a successful entrepreneurial journey. Meaning, that entrepreneurs’ stronger PsyCap controls the behavior of individuals toward achieving goals, thereby making the behavior control lead toward organization success (Zaremohzzabieh et al., 2019).

Moreover, revealing the increasing importance of PsyCap in entrepreneurship, research shows that PsyCap significantly influences business performance. The literature states that PsyCap resources harness the investment, thereby enhancing ES in the shape of constant growth (Baluku et al., 2018). The PsyCap strengthens the organizational strategic competencies, thus establishing a positive relationship between ES and PsyCap (Paul and Tresita, 2018). Consequently, the above finding provides a deep insight into the relationship between PsyCap and firms’ success.

P5: Psychological Capital is more positively related to ES.

### The Mediating Role of Psychological Capital

The rapidly changing business environment has extended the need for advanced technological developments. In the growing age of digitalization, entrepreneurial skills alone cannot drive business operations. In this context, digital knowledge enhances the skills needed for solving the firm’s problem. Technical knowledge assists entrepreneurs in the decision-making process, thereby encouraging creative thinking. The progressing significance of digital knowledge has made it critical to understand the role of technological advancements in entrepreneurship. In recent years, organizations have realized the crucial ability of technologies in improving the firms’ performance with rapid technological changes (Lei et al., 2020). The organization’s technical capability largely depends upon the generation of innovative information, processes, and products. All these elements drive the firms’ operations, leading the firm to attain ES. Together, knowledge and capability constitute the enterprise’s ability to perform (Santoro et al., 2019). The careful swift of knowledge creation to knowledge diffusion enhances the entrepreneurs’ capability to achieve business success. Indeed, the organization’s technological capabilities boost the firms’ economic performance.

The progression of digital technologies extensively facilitates knowledge development. The technical knowledge provides the firms a deeper understanding of evolving technologies, essentially gaining ES. The ES largely depends upon firms’ technical ability, allowing the firms to take advantage of the strategic market opportunities (Chen et al., 2018). The proactive behavior of PsyCap allows the entrepreneurial venture to gain market sustainability (Palazzeschi et al., 2018). Consequently, PsyCap has been fundamentally recognized as the potential force driving the firms’ technological capability (Lei et al., 2019), thereby stimulating a successful entrepreneurial journey.

Technological innovation is crucial for sustaining long-term ES. Radical innovation (i.e., technological innovation) refers to the knowledge that empowers the firm to develop innovative products, eventually achieving economic development (Nguyen et al., 2019). Radical innovation (i.e., technology) significantly improves the development of TK (i.e., radical innovation), subsequently sustaining entrepreneurial growth. The literature stresses the role of technical knowledge innovation influencing the individuals’ PsyCap. The research records that PsyCap fosters the firms’ innovation, thereby making critical contributions to the firm’s productivity (Gawke et al., 2019).

The firms’ innovation ensures the development of a solid knowledge foundation, recording an improvement in the firm’s performance. In support, the research indicates that TK causes PsyCap to influence the organization’s knowledge production (Tsai et al., 2020) through interpreting employees’ innovative behavior into the firm’s success. Fundamentally, the entrepreneurial concept has a deep root in psychology. Entrepreneurs’ prime objective is to drive market opportunities, leading the new enterprises to increase their business value. In particular, a study shows that an increase in PsyCap influences EO, subsequently improving the business performance (Moghimi Esfandabadi et al., 2015).

Psychological capital facilitates entrepreneurial innovation leading to sustainable performance. In particular, results explain that the core competence of PsyCap promotes firms’ growth and performance (Montani et al., 2020). Researchers suggest that organizations’ PsyCap attributes drive the firm performance, thus gaining business success (Muldoon et al., 2019). Significantly, EO strengthens the PsyCap leading to firm success. Successful organization formation largely depends upon the sustainability of the entrepreneurial venture. EO plays its part in enhancing the firms’ performance, thus contributing toward organization economic development. The psychological aspect of entrepreneurship strengthens the organization’s performance, thereby achieving business success. PsyCap fundamentally mediates the relationship between EO and firms’ performance. The finding indicates that firms with higher EO exhibit high performance by enhancing entrepreneurial traits (i.e., innovativeness, reactiveness, and aggressiveness) (Javed et al., 2018). Given the explanation, PsyCap refers to the individual’s mental state that enables them to exhibit motivated behavior, fostering the innovation process, thereby boosting firms’ overall performance. Perhaps, a clear understanding of PsyCap allows the firm to improve its performance, thus driving business growth. PsyCap influencing individual behavior, in turn, fosters the organizations’ performance. In support, the study shows that PsyCap empowers entrepreneurs to have confidence in firms’ performance, thus achieving business success (Zhang et al., 2020).

Perhaps, EO is a unique concept ensuring the adaption of psychological factors (e.g., risk, self-efficacy, resilience, controlled behavior), thus gaining economic sustainability. In conclusion, the study indicates that PsyCap constructs (i.e., risk, innovation, and proactive behavior) provide the firm with open opportunities, thus increasing the entrepreneurs ability to successfully run the business (Herlinawati et al., 2019).

P6: Technological Knowledge is more positively related to ES with PsyCap.

P7: Entrepreneurial Orientation is more positively related to ES with PsyCap.

**FIGURE 1:**
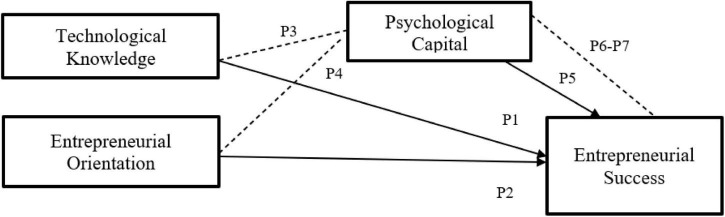
Proposed conceptual framework.

Figure 1 shows the proposed conceptual framework.

## Discussion

Entrepreneurial success is still the focus of research; this paper provides a new vision into the literature by providing a well-organized conceptual model of ES. This model has theoretically discussed the concept of ES with its perquisites and outcomes. This proposed model looks into the ES process, fundamentally incorporating the sections vital for achieving ES. The framework of this model cooperates where all the constructs are connected and reciprocally affect one another. However, each factor is a positive effect on others. The model’s prerequisites explain the factors that influence the ES process in extension TK, EO, and PsyCap.

Successful entrepreneurship ensures accelerating the firms’ TK (Belitski et al., 2021). Previous studies indicate that the relationship between TK and ES has been the subject of interest in the literature (Wach et al., 2020). Whereas there is consent that TK is an important factor for ES (Gupta and Mirchandani, 2018). Therefore, the previous studies also confirmed that EO plays a significant role in the ES (Kittikunchotiwut, 2020). This study suggested that TK significantly affects PsyCap; it can enhance the technological learning of capital resources to utilization in the job. This suggestion is similar to the existing literature (Shen et al., 2019).

Accordingly, the EO enables the firms to achieve fruitful results, thus driving the project’s success (Martens et al., 2018). Based on the previous literature and theories, this study suggests that EO significantly affects PsyCap, enhancing the use of capital resources in an entrepreneurial context (Hasan et al., 2019). The PsyCap in entrepreneurship plays a significant role in accelerating the firms’ operations, thus determining long-term business success. The potential psychological ability enhances the individuals’ commitment to the organizations’ goals, leading to a higher organizational performance which is significant for achieving the firm’s long-term success. Undoubtedly, positive PsyCap influence the firms’ performance (Hasan et al., 2020), thus preserving the business’s long-term progress. In particular, the literature shows that PsyCap has increasingly gained popularity, propelling organizations to enhance their output, thus accomplishing business success. The psychological components (e.g., resilience, efficacy, optimism) yield the organizations to foster their productivity, thereby ensuring firms’ long-term achievement (Lee and Yang, 2019). Consequently, the dimension of PsyCap makes the organizations achieve excellence (Larsson and Thulin, 2019), promoting business success. The study also suggests that PsyCap significantly affects ES, consistent with previous studies (Paul and Tresita, 2018).

This study also suggests the mediating effect of PsyCap between TK and ES. This suggestion is consistent with previous literature (Lei et al., 2020). Further, this study proposed the mediating effect of PsyCap between EO and ES. Previous studies suggest that a connection between these constructs can increase ES (Herlinawati et al., 2019).

## Conclusion

Entrepreneurship is a prime contributor integrating the novel phenomenon (e.g., PsyCap, TK, EO) into a firm’s business practices. It encourages the world economies to accelerate organizational development. Essentially, entrepreneurship is a significant marvel that drives the firms’ success. This paper develops a theoretical roadmap for future researchers’ by joining the factors that fundamentally contribute toward ES.

Entrepreneurship research broadens the research scope by fully exploring the role of TK on firms’ success. The study advances the research on the phenomenon of tech knowledge, EO, and PsyCap affecting the firms’ progress. The finding suggests technology knowledge and EO achieve business excellence, thus ensuring firms long-term progress. Moreover, the study also records a positive relationship between PsyCap and corporate success. Indeed, these positive results encourage the organizations to involve in entrepreneurship, thus gaining business success.

### Implications and Future Research

The results based on theoretical discussion and conclusion also bring implications for practice and increase the broad future directions for other researchers. A significant contribution of this paper is to propose an original relationship between prerequisites, phenomena, and consequences in ES. The proposed model shows a novel conceptualization of how these constructs may affect ES outcomes. This study enhances the literature by providing the theoretical literature of forerunners and outcomes for ES. In addition, this study is important implications for practitioners, entrepreneurs. This paper and the proposed model have various practical implications. Entrepreneurs should enhance their technical knowledge to increase their PsyCap to achieve ES. Moreover, EO can increase PsyCap to help ES. This research also gives entrepreneurs broad insights into decision-making and controls in place to generate success in various entrepreneurial activities.

This article represents the theoretical approach to understanding ES and its phenomenon. Therefore, more researchers need to understand how firms can be more dynamic based on their specific situations. This study demonstrated that a conceptual framework based on social cognitive theory; does not have sufficient consistency for the achievement of this subject. There is a need to move toward new ways and approaches to understand ES with TK, EO, and PsyCap.

## Author Contributions

The author confirms being the sole contributor of this work and has approved it for publication.

## Conflict of Interest

The author declares that the research was conducted in the absence of any commercial or financial relationships that could be construed as a potential conflict of interest.

## Publisher’s Note

All claims expressed in this article are solely those of the authors and do not necessarily represent those of their affiliated organizations, or those of the publisher, the editors and the reviewers. Any product that may be evaluated in this article, or claim that may be made by its manufacturer, is not guaranteed or endorsed by the publisher.
